# A Novel Thermal-Visual Place Learning Paradigm for Honeybees (*Apis mellifera*)

**DOI:** 10.3389/fnbeh.2020.00056

**Published:** 2020-04-15

**Authors:** Ricarda Scheiner, Felix Frantzmann, Maria Jäger, Oliver Mitesser, Charlotte Helfrich-Förster, Dennis Pauls

**Affiliations:** ^1^Behavioral Physiology and Sociobiology, Theodor-Boveri-Institute, Biocenter, University of Würzburg, Würzburg, Germany; ^2^Department of Animal Physiology, Institute of Biology, Leipzig University, Leipzig, Germany; ^3^Neurobiology and Genetics, Theodor-Boveri-Institute, Biocenter, University of Würzburg, Würzburg, Germany; ^4^Field Station Fabrikschleichach, Biocenter, Department of Animal Ecology and Tropical Biology, University of Würzburg, Würzburg, Germany

**Keywords:** learning, memory, honeybee, temperature, landmark

## Abstract

Honeybees (*Apis mellifera*) have fascinating navigational skills and learning capabilities in the field. To decipher the mechanisms underlying place learning in honeybees, we need paradigms to study place learning of individual honeybees under controlled laboratory conditions. Here, we present a novel visual place learning arena for honeybees which relies on high temperatures as aversive stimuli. Honeybees learn to locate a safe spot in an unpleasantly warm arena, relying on a visual panorama. Bees can solve this task at a temperature of 46°C, while at temperatures above 48°C bees die quickly. This new paradigm, which is based on pioneering work on *Drosophila*, allows us now to investigate thermal-visual place learning of individual honeybees in the laboratory, for example after controlled genetic knockout or pharmacological intervention.

## Introduction

Temperature is an important modality for honeybees and small variations in ambient temperature can have large effects on honeybee development and behavior (Tautz et al., 2003; Groh et al., 2004; Jones et al., 2005; Kablau et al., 2020a,b). High temperatures are particularly critical not only for the proper development of larvae but also for adult honeybee workers. When in summer the temperatures exceed a threshold of 34°C, workers move outside the hive to lower hive temperature by fanning and by carrying water inside (Lindauer, 1954). With increasing ambient temperatures due to climate change, honeybee foragers are increasingly facing extreme temperatures during their foraging bouts (Soroye et al., 2020). Cold spots in an otherwise hot environment are, for example, provided by leaves of trees, which give shadow and thus allow honeybees and other bees to relax from temperature stress (Böll et al., 2019). Individual bees try to avoid temperatures above 44°C and even respond with the extension of their sting to stimulations with heat, indicating that heat can serve as an aversive stimulus for these insects in learning situations (Junca et al., 2014). Honeybees are excellent models of associative learning and memory (Giurfa, 2003). In addition to training bees to associate an odor (neutral stimulus) with sugar water reward in the conditioning of the proboscis’ extension response (for review see Giurfa and Sandoz, 2012), they can learn to associate an odor with a short and aversive heat stimulus in aversive conditioning of the sting response such that the odor can predict a punishment by high temperature (Junca et al., 2014). Most of the aversive learning paradigms rely on the observation of sting extension, which requires that the bee is immobilized on her back in a highly unnatural position, which might induce a large degree of stress in the insect.

To overcome this problem of fixation and to ask if honeybees can actually employ a normal ambient temperature as a reward or escape stimulus in an otherwise unpleasantly hot arena, we introduce a thermal-visual place learning paradigm for honeybees, which is based on a similar arena for fruit flies (Ofstad et al., 2011). A great advantage of this novel paradigm for honeybees is that the bees can walk about freely in the arena, which is highly advantageous over the fixed situation necessary for the learning paradigms described above. Also, this paradigm allows us to study the physiological mechanisms underlying visual learning in an arena under fully controlled conditions using a simulated environment, which is very important for understanding how bees orientate and navigate.

## Materials and Methods

### Animals

All experiments were performed with honeybees (*Apis mellifera carnica*) from queen-right colonies maintained at the departmental apiary of Würzburg University. Colonies have been treated against *Varroa destructor* regularly with a sufficient time interval to experiments. Returning foragers aged between 3 and 5 weeks were collected from the hive entrance and wings were cut on the same day of the experiment or the day before, depending on the experiment, to prevent the bees from flying about. Bees were kept overnight in an incubator for 24 h at 28°C and 60% humidity. Bees could feed *ad libitum* from either 30% or 50% sugar solution until testing.

### Behavioral Experiments

Spatial learning experiments were performed in a visual heat maze arena, which was described for the first time by Ofstad et al. (2011). Bees were collected individually from the hive entrance the day before testing. Until the start of the experiment, they were maintained in small cages (11.7 cm × 8.6 cm × 7 cm) and had access to either 30% or 50% sucrose solution *ad libitum*, depending on the experiment. Cages were placed in an incubator maintained at 28°C and 60% humidity. Before the behavioral experiment, a bee was taken out of the cage and transferred in a small glass vial, in which it was immobilized on ice for approximately 5 min. Then, we had to cut off the wings to prevent the bees from flying about in the arena. After 2 min, the bee was placed into the arena and usually began to walk immediately.

We adapted the thermal-visual arena of Ofstad et al. (2011) for honeybees. The ground of the round arena was heated by a water bath. To introduce a safe spot within the arena, a defined temperature (here 25°C) can be set at a specific spot due to 64 individually addressable thermoelectric modules (Peltier elements; 23 × 23 × 4.2 mm^3^; *Q* = 13.1 W, *I* = 2.7 A, *U* = 8.1 V, dT = 71 K; JenMechanik Limited, Jena, Germany, see Supplementary Material). We placed thermal conductive pads and a white PVC disk (material thickness of 0.5 cm) on top of the Peltier array providing a flat and even surface for the bees. The arena was limited by an 8 mm high, 18.5 cm diameter aluminum ring heated at 60°C covered with glass disc preventing the bees from escaping. The arena was surrounded by an LED screen of 30 cm heights with a diameter of 32 cm. The LED screen consisted of twelve P4 soft modules with 256 per 64 LEDs (Shenzhen UNIT LED Company Limited, China)^1^ which was used to produce landmarks in the form of black horizontal, vertical and diagonal stripe patterns in front of a white background. Each bar covered 15° of the screen seen from the center of the arena which corresponds to a spatial frequency of 0.033 cycles/°. During the experiment, the arena was illuminated with infrared light and bees were recorded with a non-chromatic camera (DMK27BUP031 with a TCL 1216 5MP objective; The Imaging Source Europe GmbH, Germany) supplemented by an infrared transmission filter. The camera was placed perpendicular above the arena center at a height of 56 cm. Movies were recorded using IC Capture 2.4.642.2631.

The experiment aimed to show that bees can find a safe spot by use of landmarks that were projected on the LED screen. A learning session consisted of six (Experiment 1) or 10 (all other experiments) training trials of 5 min each and a subsequent test without a safe spot (apart from Experiment 1). In each trial, the safe spot and the landmark were shifted by 90° clockwise or anticlockwise, i.e., one out of four quadrants that virtually divided the arena. Thus, the relation between landmarks and safe spots always remained constant. In each trial, the time was measured until the bee reached the safe spot, where it usually remained until the safe spot was re-located and thus the place became too hot for the bee.

Movies were compressed using the program any2ufmf. Subsequently, the walking path of a bee was tracked using Ctrax 0.5.11 (Branson et al., 2009). The location of the bee and the time it needed to reach the safe spot were subsequently calculated using a custom-made R-script (see Supplementary Material). We determined the duration until the safe spot was reached for each bee in each trial. In the subsequent test, we measured the time the bee spent in the quadrant where the safe spot had been in the trial before. However, the landmark was shifted as before. We only tested one shape in our experiments, because we could not detect any preference of the bees for a landmark without a safe spot (data not shown). But, we are convinced that honeybees do not have a natural preference for this highly artificial shape because it has been demonstrated that honeybees prefer flower-like patterns and shapes (Lehrer et al., 1995). We calculated a learning index as the quotient of the time the bee spent in the correct quadrant and the time it spent in the quadrant across the correct quadrant (Ofstad et al., 2011).

### Statistics

Data were analyzed for normal distribution using the Shapiro–Wilk Normality test. The effect of training trial and treatment on the time needed to reach the safe spot was compared between different groups using repeated-measures analysis of variance (ANOVA RM, factor training trial or factor treatment, SPSS, IBM). To test whether the learning index differed from zero, one sample *T*-tests were performed (because all data appeared to be normally distributed). The learning indices of two different groups were compared using independent *T*-tests. All tests were two-tailed.

## Results and Discussion

Despite their minute brain, honeybees have impressive navigational skills, which allow them to locate diverse food sources within a range of several kilometers around their nest and to return fast and direct to their hive (Beekman and Ratnieks, 2000). Many experiments suggest that they rely on a cognitive map similar to humans (Menzel et al., 2005; Moser et al., 2008). When young bees leave the hive for the first time, they need to acquire information on the local area and perform 2 days of orientation flights before they begin to forage. If they are displaced during this time, many of them are unable to return to their hive (Capaldi and Dyer, 1999). Once they have performed their orientation flights, however, they can be displaced during their outward foraging trip and will still return to their hive using the most direct route, which often involves novel short cuts. Honeybees use both path integration, relying on a celestial compass and an odometer (Srinivasan, 2015) and view-based navigation, i.e., comparing memorized panoramic views with current views (Towne et al., 2017) for orientation in complex landscapes. Intriguingly, we still know very little about the neuronal mechanisms underlying these complex navigational skills in honeybees (Zwaka et al., 2019). Several studies indicate an important role for the mushroom bodies in honeybee orientation—a region of the insect brain involved in sensory integration and memory (Plath et al., 2017; Zwaka et al., 2019). Further, the central complex receives orientation and spatial information and processes how the bee is orientated in relation to its environment using visual working memory (Plath et al., 2017). An unresolved open question is whether there is a connection between the mushroom bodies and the central complex. Similar to vertebrates, the transcription factor *early growth response protein* 1 seems to play a role in orientation, since its expression is upregulated following a single orientation flight in the mushroom bodies in young foragers (Lutz and Robinson, 2013). A homolog of this gene is known to play a role in memory consolidation by promoting structural neuroplasticity in the brain following exposure to novel stimuli in vertebrates (Knapska and Kaczmarek, 2004).

**Figure 1:**
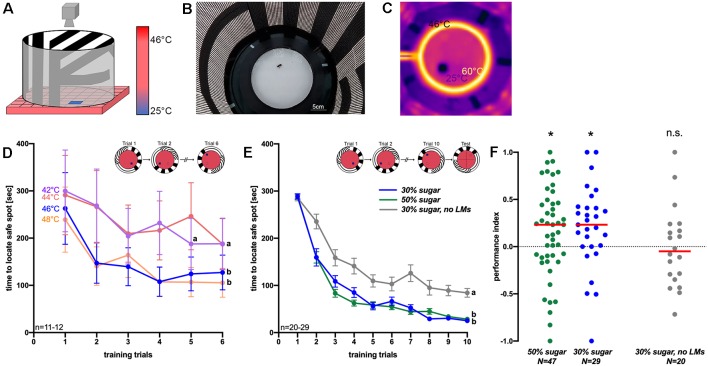
**(A)** Schematic picture of the arena for thermal visual place learning in honeybees (adapted from Ofstad et al., 2011). The ground of the arena is unpleasantly hot and only a small safe spot is (25°C) is offered to walking honeybees. The visual panorama changes with the cold tile. **(B)** Photograph of the arena from the top. Light-emitting diodes illuminate the arena from the wall. **(C)** Thermal image of the arena: spatial learning experiments were performed using 46°C ground temperature. Also, a heated ring (~60°C) and a glass lid on top prevent bees from escaping. **(D)** The time needed to reach the safe spot at different ground temperatures (42°C–48°C) within in six training trials of 5 min each. Different letters (a,b) indicate significantly different groups (*n* = 11–12). **(E)** Times to reach the safe spot using 46°C ground temperature. Of bees that could use landmarks (green and blue graphs; bees of these two groups received different sugar solutions before training) and those which did not have landmarks (gray graph) within 10 training trials. The inlet shows examples of the safe spot position. In **(D,E)**, mean values and standard errors are shown (*n* = 20–29). **(F)** Performance indices of groups trained without landmarks (gray dots; *n* = 20) and of those with landmarks [green (*n* = 47) and blue dots (*n* = 29)] which differed in the sugar concentration they could feed on before training (green: 50% and blue: 30%). *P < 0.05, *T*-test; n.s., not significantly different from zero (LM, Landmarks).

To study visual place learning under controlled laboratory conditions for future analyses on the molecular mechanisms and genetics underlying learning and navigation, we established a thermal visual arena for honeybees, which was adapted from an arena for fruit flies (Ofstad et al., 2011). It was inspired by the Morris water maze for rodents and heat mazes for crickets and cockroaches (Morris, 1981; Mizunami et al., 1998; Wessnitzer et al., 2008; Ofstad et al., 2011). In this arena, honeybees learn to escape an unpleasantly hot environment (>44°C) by approaching a safe spot (i.e., a 25°C cool tile) associated with a visual stimulus (Figures 1A–C).

Honeybees are very sensitive to ambient temperature and maintain a brood nest temperature between 32°C and 36°C, with an optimal temperature of 35°C, to support the appropriate brood development (Tautz et al., 2003). When adult honeybees were tested for their thermotaxis on an aluminum block with a temperature gradient of 28–48°C, they preferred temperatures of 34–35°C (Kohno et al., 2010). At the individual level, bees avoid temperatures above 44°C and respond with a sting extension to heat stimulations (Junca et al., 2014). The lethal temperature for honeybees (*Apis mellifera carnica*) is at around 50°C (Kovac et al., 2014). We, therefore, applied temperatures between 42°C and 50°C to test which temperature is optimal to induce avoidance of honeybees in our arena.

The only visual cues for spatial orientation for the bee in the arena are provided by the surrounding LED panorama (Figures 1A–C). This displays three different stripe patterns with vertical, horizontal and diagonal bars (Figures 1A,B; Ofstad et al., 2011). To assess visual place learning, an individual honeybee is introduced into the arena. During training, we measure the time which the bee needs to reach the safe spot within a time window of 5 min. A surrounding heated ring (~60°C) and a glass lid prevent the bee from flying off the arena (Ofstad et al., 2011). Once the bee has located the safe spot, it remains there until the cool tile and the corresponding visual panorama are rotated randomly clockwise or anticlockwise by 90°. Then the bee starts searching for the safe spot anew. With increasing training trials the bees associate the position of the safe spot relative to that of the visual landmark. Temperatures between 42°C and 50°C (ground temperature) were applied in a first experiment in six trials to investigate at which temperature the bees find the safe spot fastest with repeated training. At 50°C, five out of seven individuals died after 5 min in the arena, so that this temperature was abandoned. At the other temperatures (42°C, 44°C, 46°C and 48°C), honeybees became increasingly faster in locating the safe spot with each training trial (Figure 1D). The two higher temperatures led to a particularly steep decrease in time to reach the safe spot. For that reason, we selected 46°C (second-highest temperature; at 46°C 20% of bees died during training, mostly because they did not accept the safe spot) for further experiments, when asking if bees would use the visual landmarks for orientation. In this experiment, one group of bees (Figure 1E; blue line) could use the T shaped landmark (border area between horizontal and vertical stripes) in the arena, which changed its relative position together with the safe spot (Figure 1E, inset). A second group of bees did not get any landmarks (the screen was set off; Figure 1E, gray graph). Further, we increased the number of training trials to ten, because after six trials not all of the groups tested before appear to have reached an asymptotic performance level. Although both groups showed a significant decrease in time to reach the safe spot (Figure 1E; effect of training trial: *F*_(6,327)_ = 85.86; *P* < 0.001, ANOVA RM), the bees which could employ landmarks showed a significantly stronger reduction in the time to reach the safe spot (effect of landmark presence: *F*_(1,49)_ = 354, *P* < 0.001).

Immediately after training, the bees faced a probe trial without a safe spot to test for visual place memory expression (described in Ofstad et al., 2011). The landmark changed its position as before. We hypothesized that bees should spend significantly more time in the quadrant of the arena where the visual landmark suggests the safe spot to be, even though there is no safe spot to escape the still high temperature in the arena during testing. The performance index of bees, i.e., the time bees spend in the target quadrant (the quadrant with the safe spot to be) in comparison to the opposing quadrant (Ofstad et al., 2011), was significantly larger than zero (Figure 1F; *T* = 2.17, *P* < 0.05, *n* = 47, for 50% sugar and *T* = 2.51, *P* < 0.05, *n* = 29, for 30% sugar), indicating place memory expression. In the absence of landmarks, however, the performance index did not differ from zero (Figure 1F; *T* = 0.1; *P* = 0.91, *n* = 20).

In many experiments on learning in honeybees [but also in other insects such as *Drosophila* and rodents (Lukoyanov et al., 2002; Friedrich et al., 2004; Krashes et al., 2009)], the feeding status of the individual has a strong effect on the behavioral response and in particular on learning performance. In honeybees, associative appetitive learning performance, for example, strongly depends on individual sucrose responsiveness (Scheiner et al., 1999, 2001a,b, 2005, 2013) and starvation time (Friedrich et al., 2004), which affects sucrose responsiveness. A starvation period of 18 h before training leads to a significantly higher memory performance than a starvation period of 4 h (Friedrich et al., 2004). Similarly, a high sugar water concentration (30%) used as a reward leads to a significantly higher acquisition performance than a low sugar concentration of 1.6% (Scheiner et al., 2005). Bees with a high sucrose responsiveness, i.e., good learners, also display a higher brain activity of cAMP-dependent protein kinase (PKA; Scheiner et al., 2003) and have higher levels of the biogenic amine octopamine (Behrends and Scheiner, 2012). Both PKA and octopamine play important roles in associative appetitive learning and memory formation (Fiala et al., 1999; Scheiner et al., 2006) and may have a similar function in thermal-visual place learning in honeybees.

Accordingly, we hypothesized that place memory expression in bees is dependent on the feeding status, as food deprivation at the individual or colony level is the main driving force for foraging behavior (Seeley, 1989; Desmedt et al., 2016). We, therefore, asked if learning in a thermal-visual arena may be affected by the sugar concentration which the bees had access to before training. Bees fed with either 30% or 50% *ad libitum* sugar solution before training displayed a significant decrease in time to reach the safe spot (effect of training trial: *F*_(9,684)_ = 209.32; *P* < 0.001, ANOVA RM), while the time to locate the safe spot of both groups did not differ statistically (factor feeding status: *F*_(1,76)_ = 0.68; *P* = 0.411, ANOVA RM). During the test, the performance index of both groups differed significantly from zero (30%: *T* = 2.51, *n* = 29, *P* < 0.05; 50%: *T* = 2.17, *n* = 47, *P* < 0.05) suggesting place memory expression. The performance index of the two groups receiving different sugar solutions did not differ significantly (*T* = 0.50; *P* > 0.05).

Many similar setups exist to test spatial orientation and learning in various insects including honeybees, fruit flies, ants, crickets and cockroaches (Zhang et al., 1996; Mizunami et al., 1998; Schatz et al., 1999; Menzel et al., 2005; Neuser et al., 2008; VanderSal, 2008; Wessnitzer et al., 2008; Foucaud et al., 2010; Ofstad et al., 2011; Tsvetkov et al., 2019). Many of the available paradigms investigating spatial learning in honeybees rely on food rewards. Tsvetkov et al. (2019), for example, developed a food search task adopted from the vertebrate literature for honeybees to study spatial learning. In their assay, the bees have to learn the location of artificial flowers inside the testing arena. Other paradigms use the successful return to the hive or the feeder after displacement as rewards (Menzel et al., 2005). Still, other paradigms employ complex mazes to study spatial learning and memory in bees (Zhang et al., 1996). Our new assay employing visual place learning in an arena under fully controlled conditions relies on temperature as a reinforcer. It offers several advantages, including the possibility of high-resolution analysis of the individual behavior, during training and testing. The recording of single animals allows a detailed and individual analysis including parameters such as quadrant distribution and transitions, speed, and directional changes when using a suitable tracking software like CTRAX (in this study; Branson et al., 2009) or others (Pérez-Escudero et al., 2014; Werkhoven et al., 2019). Ofstad et al. (2011) showed no difference in place learning performance between individually tested flies and flies tested in groups. Whether this also applies to social animals such as the honeybee will have to be tested in the future (Howard et al., 2019).

Taken together, our results show that honeybees can rely on visual landmarks to locate and learn a safe spot position in an otherwise hot arena. Further, the bee’s performance in this non-food reinforced task is independent of the feeding regime, at least for the selected standard sugar solutions (30% and 50%) given to the bees before the day of training. Our paradigm allows us now to study navigational skills of individual honeybees under controlled laboratory conditions, enabling manipulations and intervention with neuronal signaling pathways to understand the neuronal mechanisms underlying visual navigation and learning. The arena also allows detailed comparative studies, as different model organisms such as fruit flies, honeybees, but also ants can be trained with the same quality of stimuli (light and temperature) under controlled laboratory conditions. It may also provide a link to vertebrate studies because setups like the Morris Water Maze or the Radial Arm Maze have been used there for many years to study place learning and spatial tasks related to both basic and applied, clinical research (Savonenko et al., 2005; Vorhees and Williams, 2006; Wolf et al., 2016). Alzheimer’s disease, for example, could serve as an example here. To understand the disease in its entirety and to develop possible effective treatment strategies, mouse models are tested in different spatial memory tasks to characterize the cognitive profile. A major opportunity is therefore to identify fundamental mechanisms of memory formation and memory loss in insects using a similar behavioral assay like the visual place memory arena described in this study and in Ofstad et al. (2011).

## Data Availability Statement

The datasets for this study and further details on the arena are available from the authors upon request.

## Author Contributions

RS, FF, CH-F, and DP conceived and designed the experiments. RS, MJ, OM, and FF performed the experiments. RS, MJ, FF, OM, CH-F, and DP analyzed the results. RS and DP wrote the article. All authors provided comments and approved the manuscript.

## Conflict of Interest

The authors declare that the research was conducted in the absence of any commercial or financial relationships that could be construed as a potential conflict of interest.

## Footnotes

^1^www.ledcontrolcard.com
