# Supplementary material for: A Novel Thermal-Visual Place Learning Paradigm for Honeybees (Apis mellifera)
Source: Front Behav Neurosci. 2020 Apr 15;14:56. doi: 10.3389/fnbeh.2020.00056 (PMC7174502; doi:10.3389/fnbeh.2020.00056)
Supplement: Supplementary file 1 [file Data_Sheet_1.PDF]

Supplementary Information

A novel thermal-visual place learning paradigm for honeybees (*Apis mellifera*)

Scheiner et al.

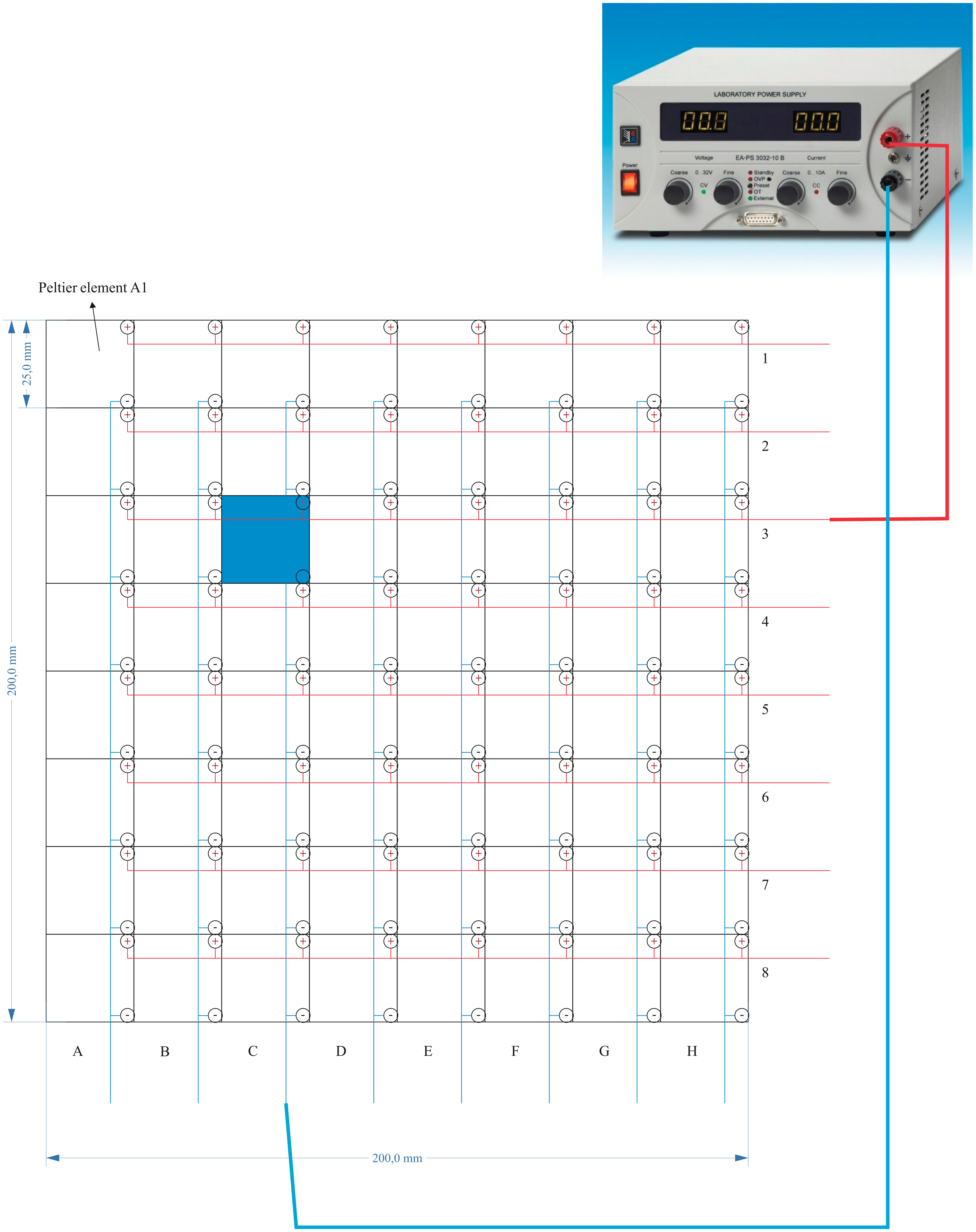
LABORATORY POWER SUPPLY
Voltage
EA-PS 3032-10 B
Current
Power
Coarse
0...32V
Fine
CV
Standby
OVP
Preset
OT
External
Coarse
0...10A
Fine
CC
Peltier element A1
25,0 mm
200,0 mm
1
2
3
4
5
6
7
8
A
B
C
D
E
F
G
H
200,0 mm

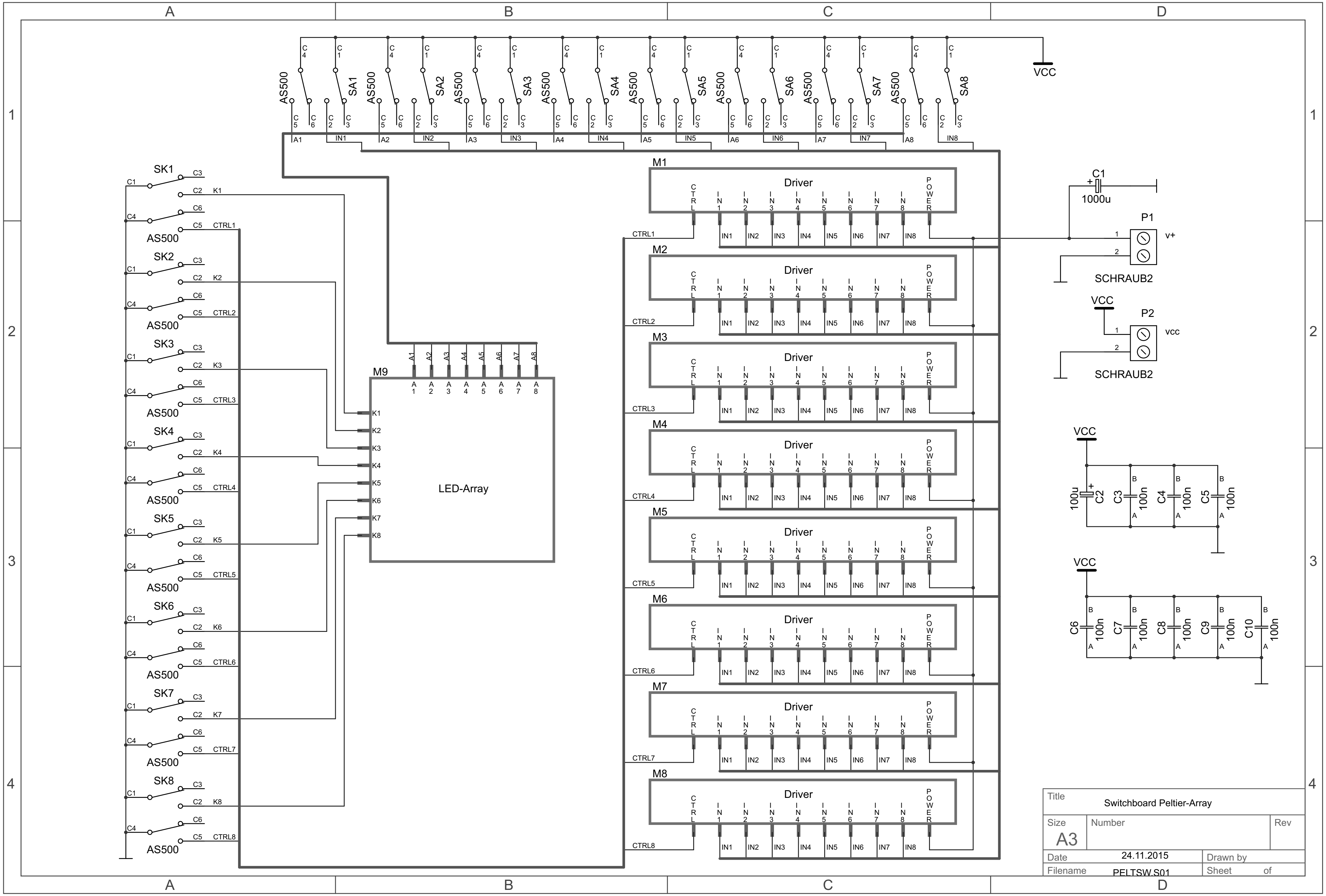

A
B
C
D
1
2
3
4
VCC
SA1
SA2
SA3
SA4
SA5
SA6
SA7
SA8
AS500
A1
A2
A3
A4
A5
A6
A7
A8
IN1
IN2
IN3
IN4
IN5
IN6
IN7
IN8
SK1
SK2
SK3
SK4
SK5
SK6
SK7
SK8
C1
C2
C3
C4
C5
C6
K1
K2
K3
K4
K5
K6
K7
K8
CTRL1
CTRL2
CTRL3
CTRL4
CTRL5
CTRL6
CTRL7
CTRL8
M1
M2
M3
M4
M5
M6
M7
M8
M9
Driver
CTRL
POWER
LED-Array
C1
1000u
P1
v+
SCHRAUB2
P2
vcc
100u
C2
C3
C4
C5
C6
C7
C8
C9
C10
100n
Title
Switchboard Peltier-Array
Size
A3
Number
Rev
Date
24.11.2015
Drawn by
Filename
PELTSW.S01
Sheet
of

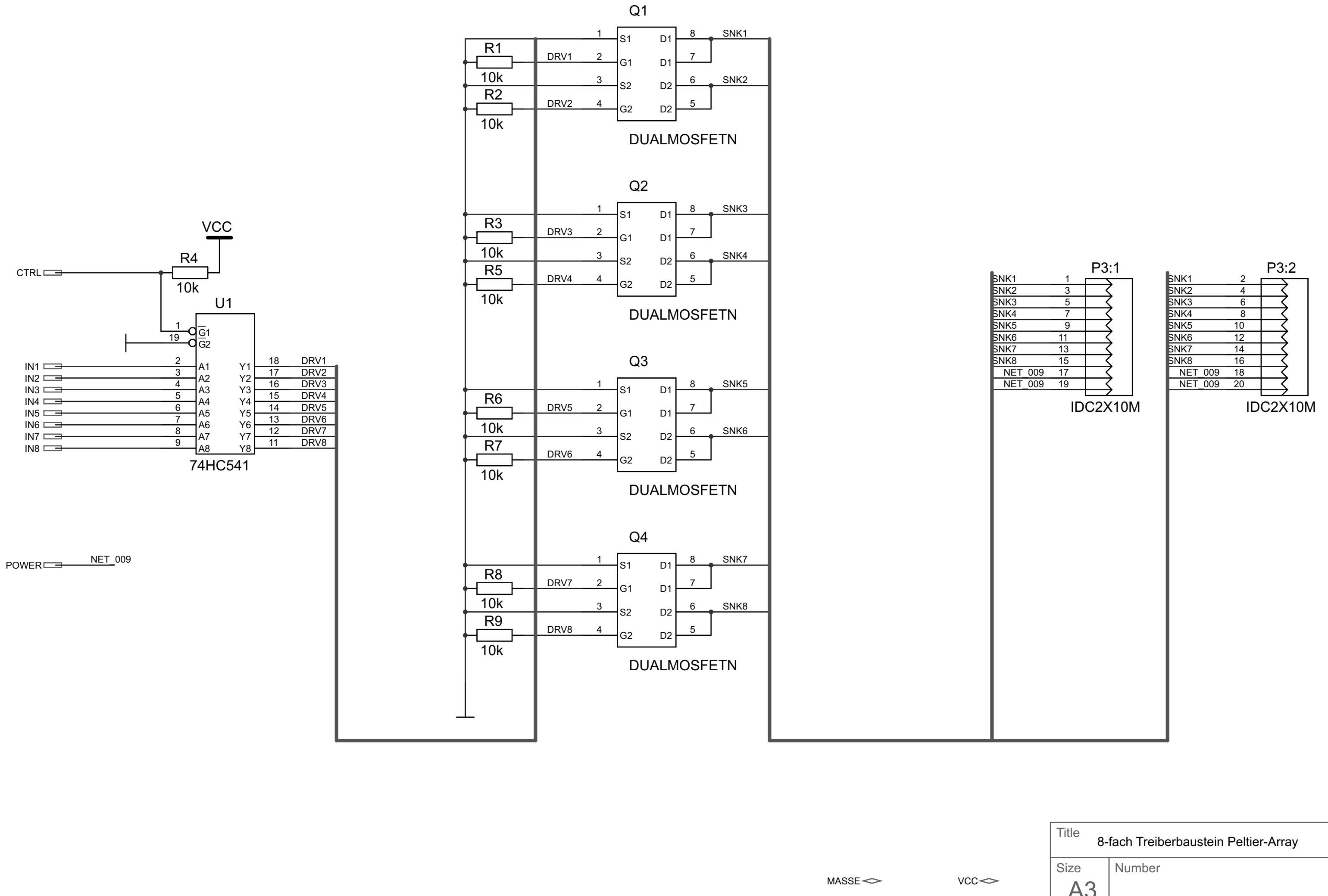

Title	8-fach Treiberbaustein Peltier-Array
Size	A3
Number	
Rev	
Date	24.11.2015
Drawn by	
Filename	PELTSW3.S01
Sheet	of

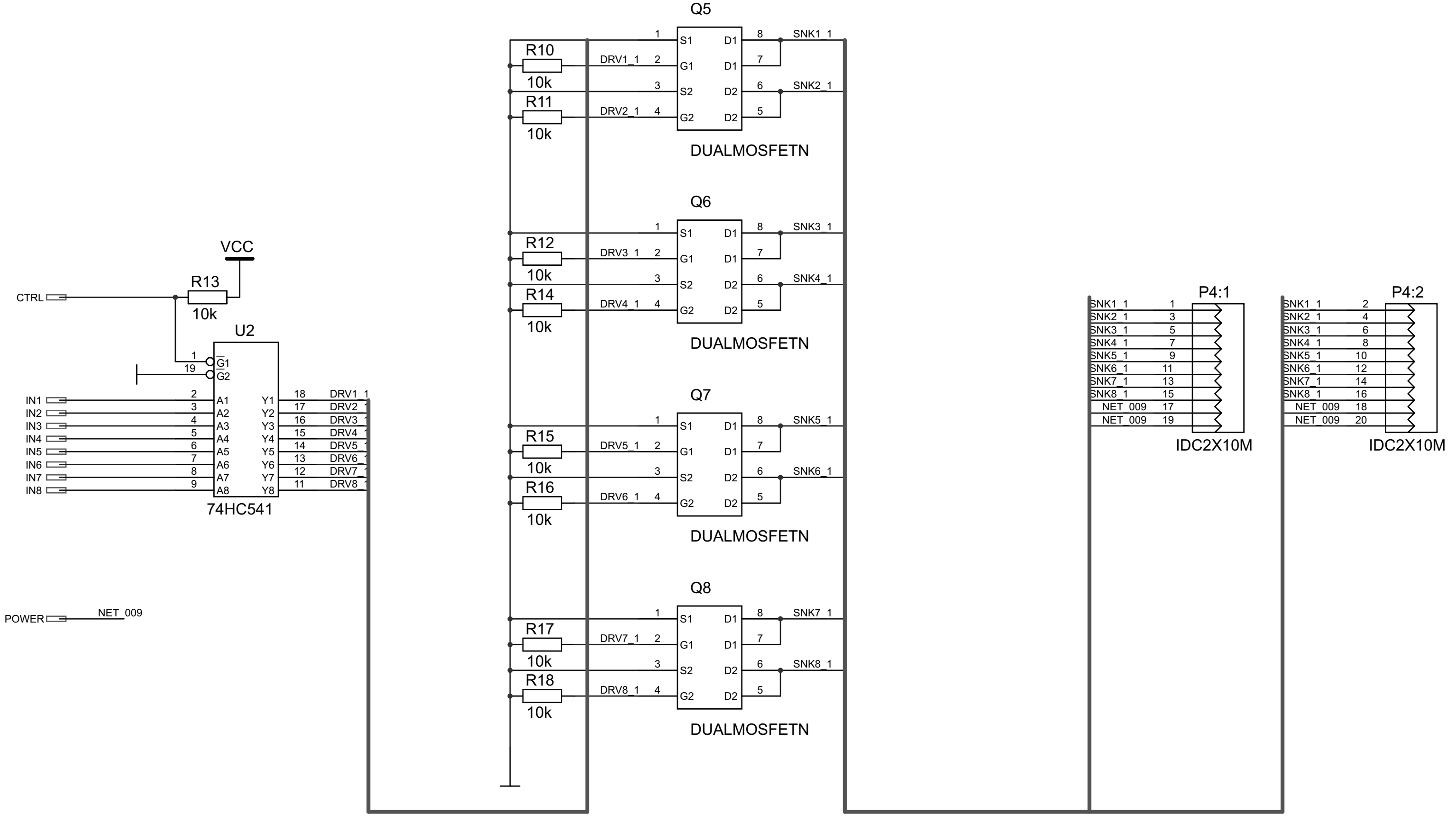

Title	8-fach Treiberbaustein Peltier-Array
Size	A3
Number	
Rev	
Date	24.11.2015
Drawn by	
Filename	PELTSW3.S01
Sheet	of

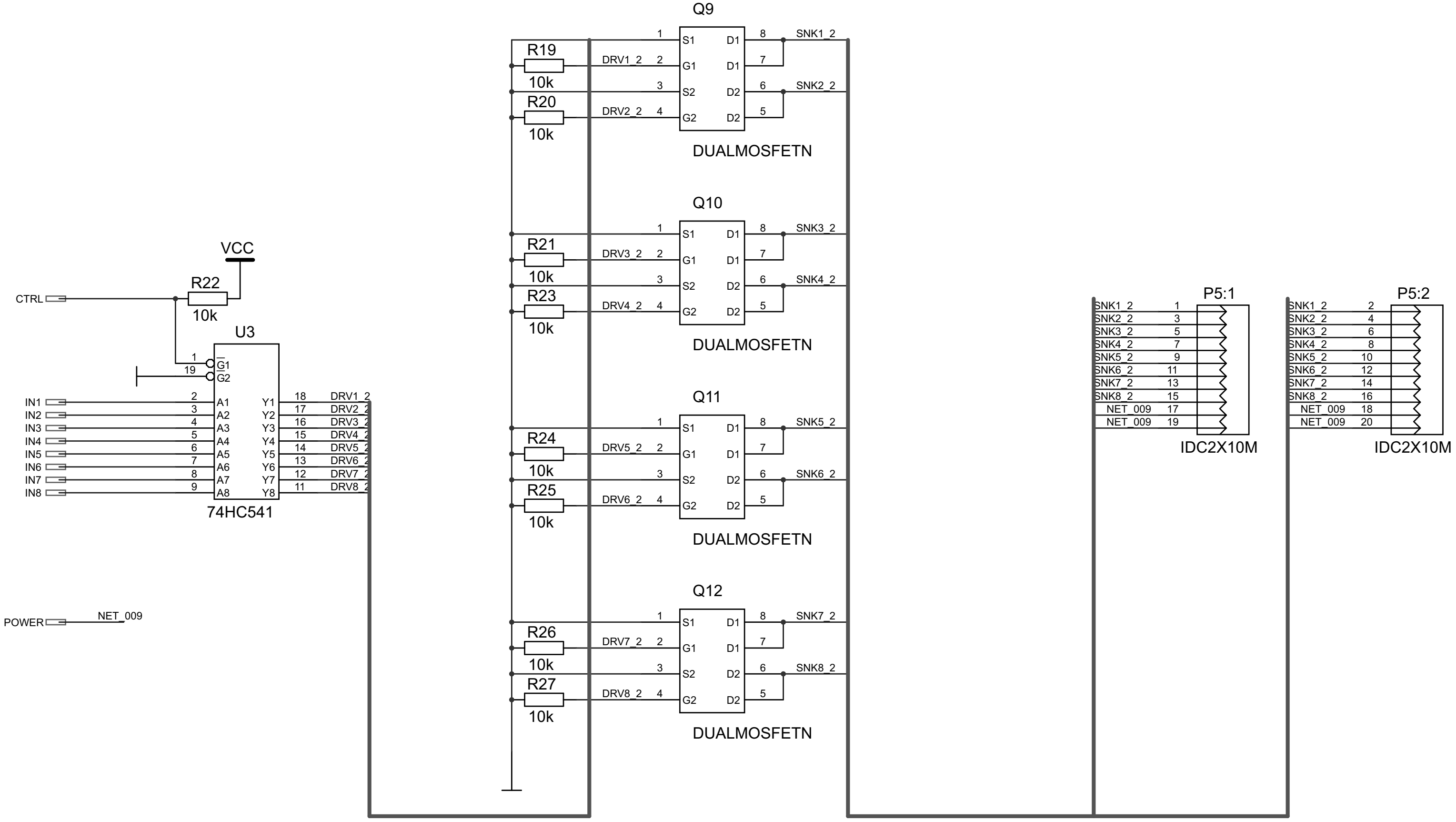

Title	8-fach Treiberbaustein Peltier-Array		
Size A3	Number		Rev
Date 24.11.2015		Drawn by	
Filename PELTSW3.S01		Sheet	of

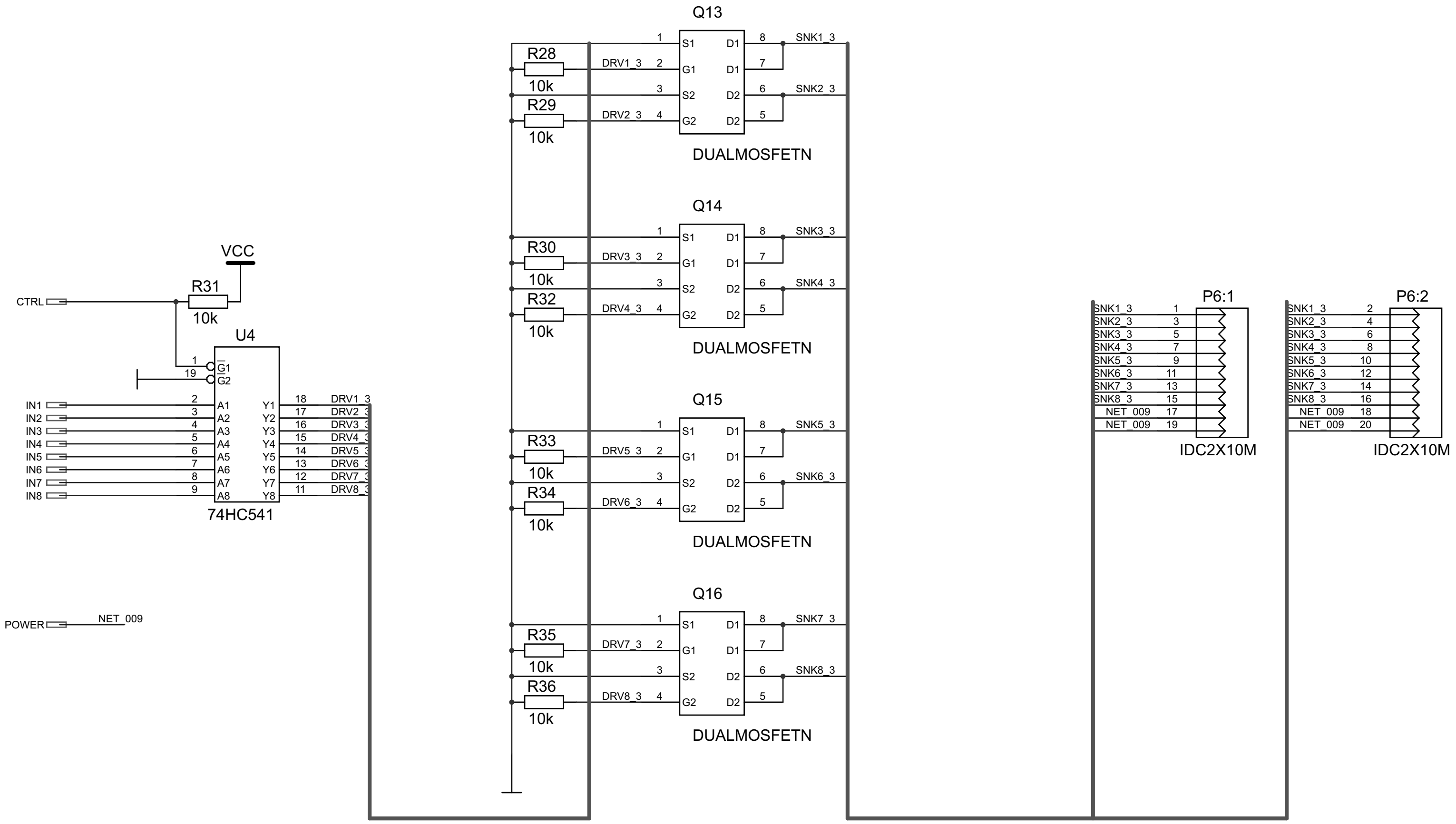

MASSE VCC

Title	8-fach Treiberbaustein Peltier-Array	
Size A3	Number	Rev
Date 24.11.2015		Drawn by
Filename PELTSW3.S01		Sheet of

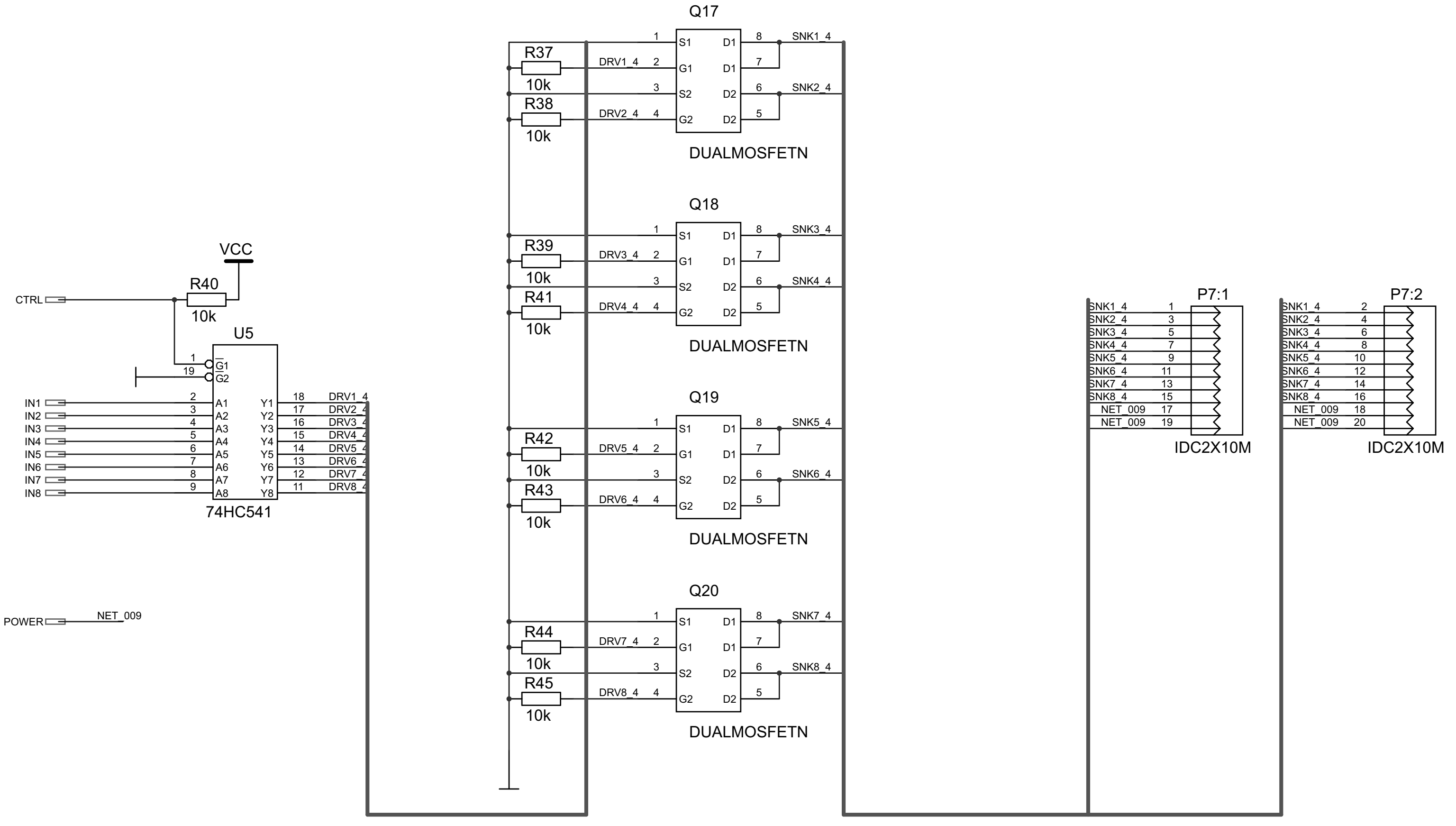

MASSE
VCC

Title: 8-fach Treiberbaustein Peltier-Array		
Size: A3	Number	Rev
Date: 24.11.2015	Drawn by	
Filename: PELTSW3.S01	Sheet	of

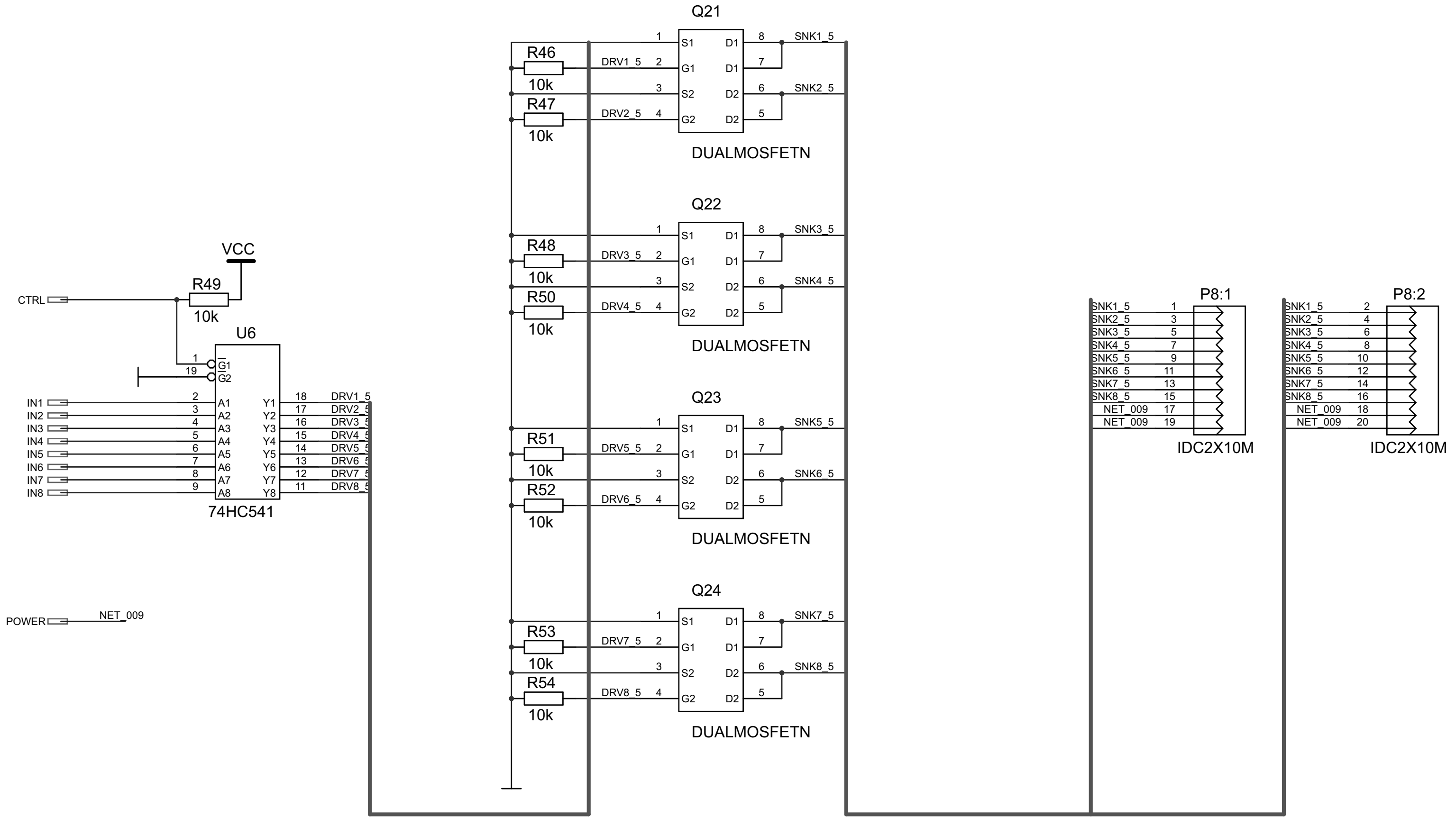

MASSE
VCC

Title	8-fach Treiberbaustein Peltier-Array	
Size A3	Number	Rev
Date 24.11.2015	Drawn by	
Filename PELTSW3.S01	Sheet of	

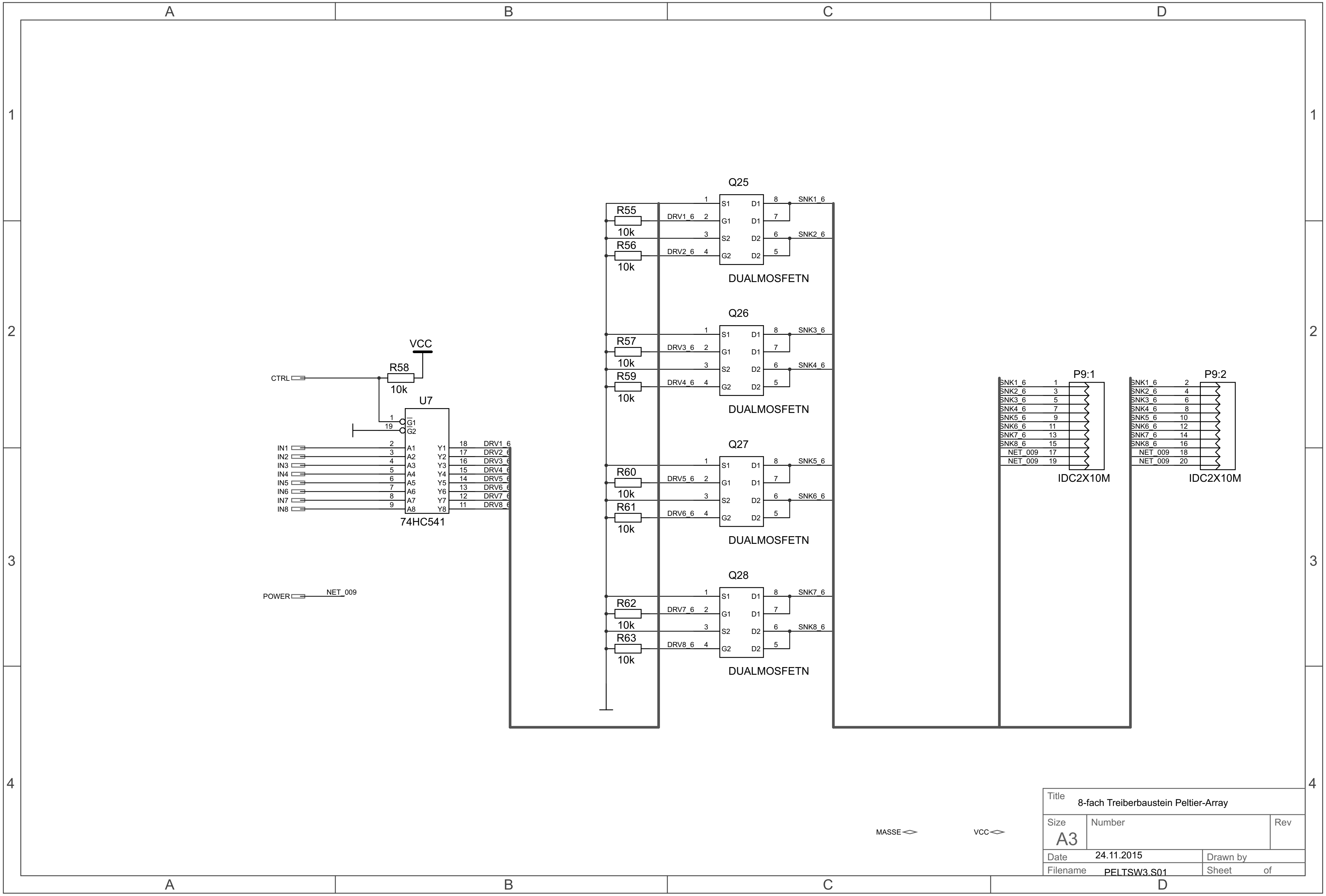
A
B
C
D
1
2
3
4
VCC
R58
10k
CTRL
U7
74HC541
G1
G2
IN1
IN2
IN3
IN4
IN5
IN6
IN7
IN8
A1
A2
A3
A4
A5
A6
A7
A8
Y1
Y2
Y3
Y4
Y5
Y6
Y7
Y8
DRV1_6
DRV2_6
DRV3_6
DRV4_6
DRV5_6
DRV6_6
DRV7_6
DRV8_6
POWER
NET_009
Q25
Q26
Q27
Q28
R55
R56
R57
R59
R60
R61
R62
R63
10k
S1
G1
S2
G2
D1
D2
DUALMOSFETN
SNK1_6
SNK2_6
SNK3_6
SNK4_6
SNK5_6
SNK6_6
SNK7_6
SNK8_6
P9:1
P9:2
IDC2X10M
MASSE
VCC
Title
8-fach Treiberbaustein Peltier-Array
Size
A3
Number
Rev
Date
24.11.2015
Drawn by
Filename
PELTSW3.S01
Sheet
of

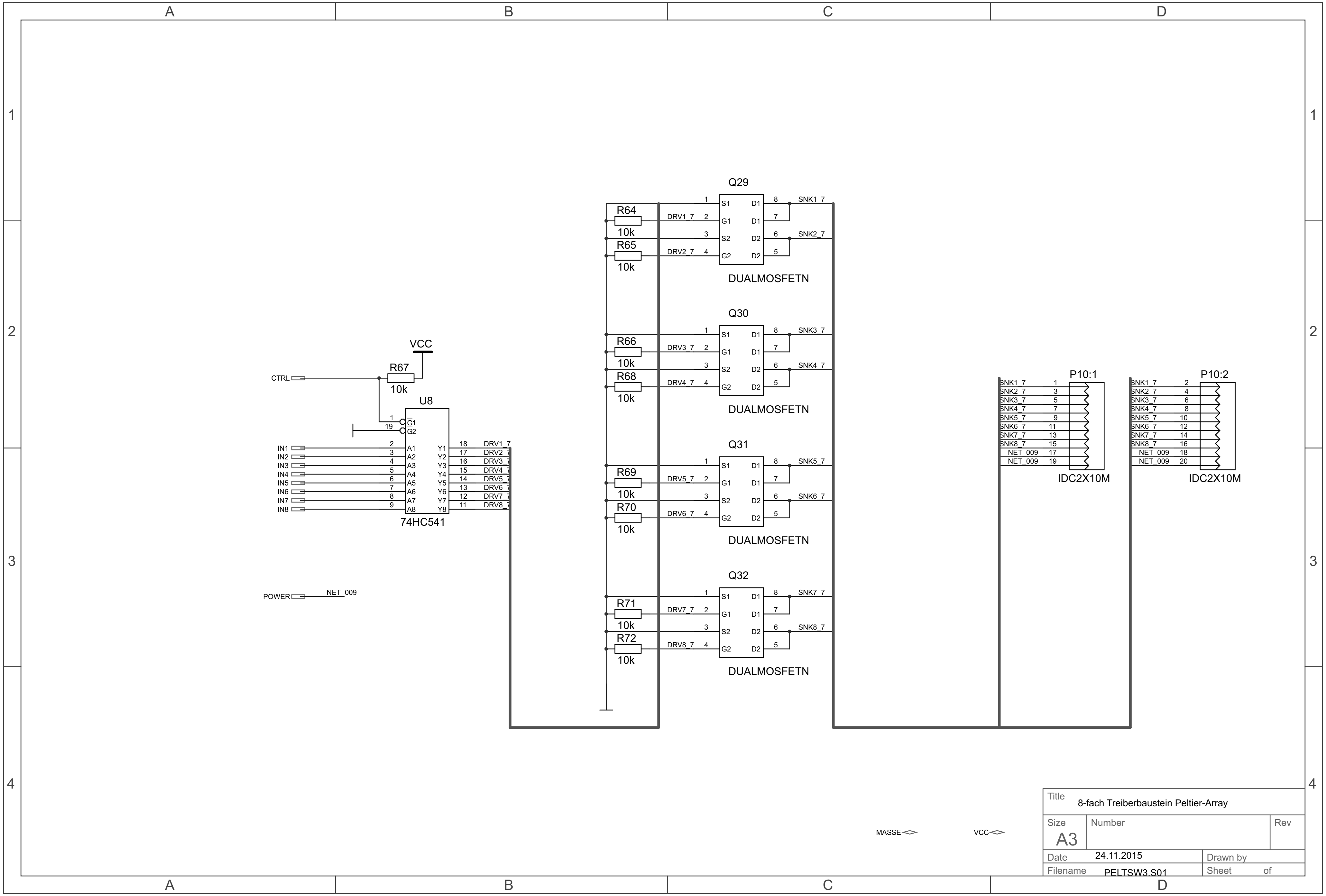

Title	8-fach Treiberbaustein Peltier-Array		
Size A3	Number		Rev
Date	24.11.2015	Drawn by	
Filename	PELTSW3.S01	Sheet	of

A	B	C	D

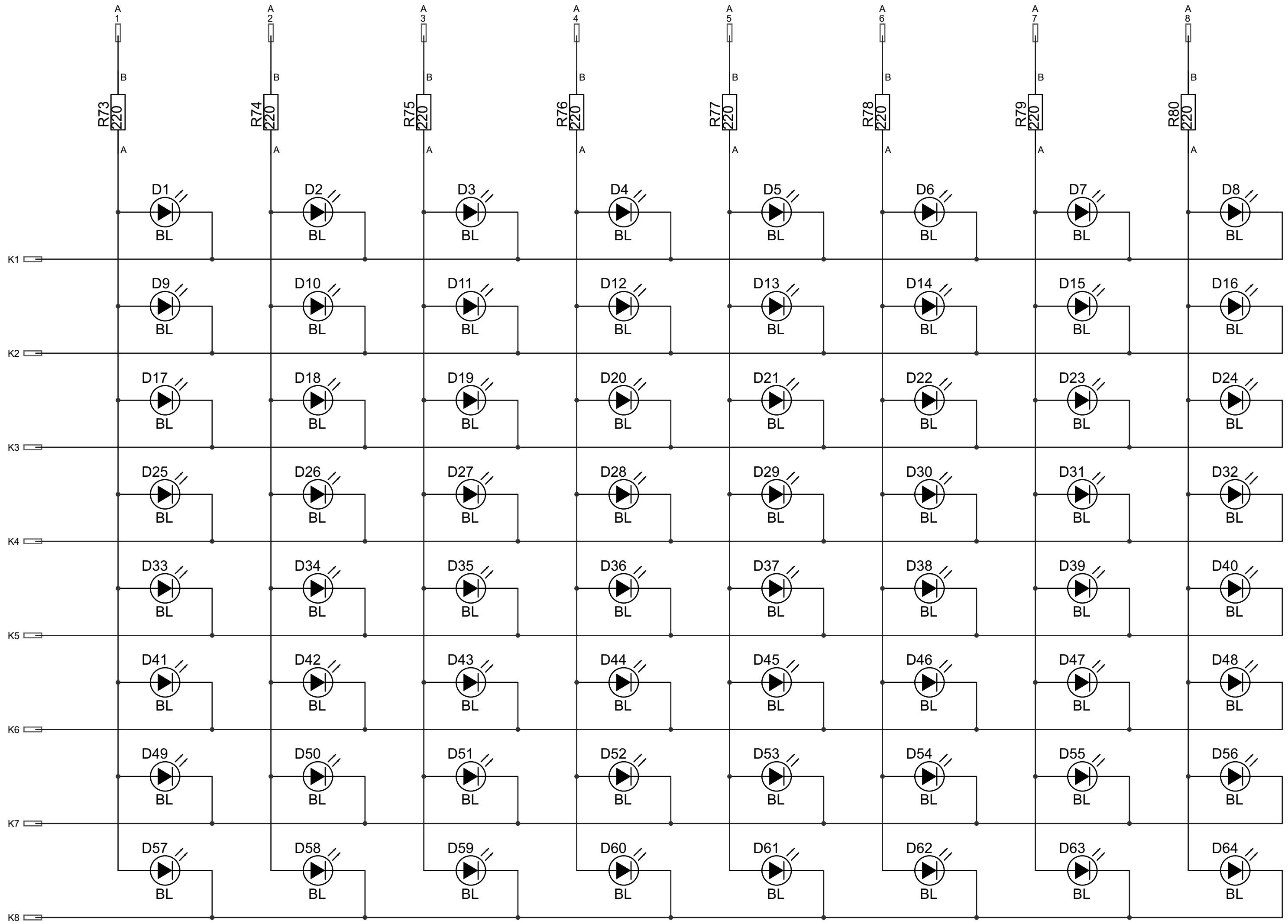

Title	LED-Array Display Peltier-Array		
Size A3	Number		Rev
Date	24.11.2015	Drawn by	
Filename	PELTSW2.S01	Sheet	of

A	B	C	D

```
# Calculate track characteristics from Ctrax data
# for Ctrax see: Ctrax: The Caltech Multiple Walking Fly Tracker
# http://ctrax.sourceforge.net/
# Main characteristic: sector assignment
# for different insects
# for different time windows (complete, 30, 60, 120, 30-90 sec.)
# Last updated: 10.3.2020, 28.06.2017
# By: Felix Frantzmann, Oliver Mitesser

# initialize data frame for output
d<-data.frame()

####################################################################
# Read time series of positions from file
# file format: Ctrax
####################################################################

# read data file
z=read.table(file=file.choose() , header=F, sep=";", dec=".")

####################################################################

# set file for R terminal output
sink("RTerminalOutput.txt")

# define center and radius of arena in pixel coordinates
# resolution 1296x972
r0=481
cx=644
cy=481

# position cold spot
# rectanle upper right
RORx=514
# transform to R:
RORy=972-602
# rectangle upper left
ROLx=398
# transform to R:
ROLy=972-602
# rectangle lower left
RULx=398
# transform to R:
RULy=972-714
# rectangle lower right
RURx=514
# transform to R:
RURy=972-714

# calculate data characteristics
# number of columns in data frame
n=length(z[1,])
# number of lines in data frame
m=length(z[,1])
```

```
# initialize vector of fly ids
flyindex=c(0)

# define pdf file for graphical output
pdf("RGraphicalOutput.pdf")

# repeat for each fly
for (i in flyindex){

  # initialize sector assignment vector for positions
  sec=rep(4,length(z[,1]))

  # set starting column for current fly
  id=i*6+1

  # calculate position angles of current track positions in polar
coordinates
  ang=atan2(z[,id+2]-cy,z[,id+1]-cx)
  ang[ang<0]=ang[ang<0]+2*pi

  # determine vector of sector for all position angles
  sec[ang > 0 & ang < (pi/2)]=2
  sec[ang > (pi/2) & ang < pi]=1
  sec[ang > pi & ang < (3/2*pi)]=4
  sec[ang > (3/2*pi) & ang < 2*pi]=3

  # determine if fly position is outside of cold spot
  raus=((z[,id+1]<rep(RULx,m)) | (z[,id+1]>rep(RURx,m)) |
(z[,id+1]>rep(RULx,m)) & (z[,id+1]<rep(RURx,m)) &
(z[,id+2]<rep(RURy,m)) | (z[,id+1]>rep(RULx,m)) &
(z[,id+1]<rep(RURx,m)) & (z[,id+2]>rep(RORy,m)))

  # determine when spot was left for the first time
  zuerstrausCS=min(which(raus==1))


###################################################################
  # Output diagnostics

###################################################################
  print(i)
  print("No. of frame after leaving the CS")
  print(zuerstrausCS)

  # vector of starting time steps for different time intervals
  sttoffvec=c(0,0,0,0,1080,1080)
  # vector of ending time steps for different time intervals
  endoffvec=c(1080,2160,3240,4320,2160,3240)

  # matrix to collect sector statistics
  ymat=matrix(ncol=12, nrow=length(sttoffvec)+1)

  # calculate first entering for each of the 4 sectors
```

```
  print("first in 1"); zuerstin1=min(which(sec==1));
print(zuerstin1)
  print("first in 2"); zuerstin2=min(which(sec==2));
print(zuerstin2)
  print("first in 3"); zuerstin3=min(which(sec==3));
print(zuerstin3)
  print("first in 4"); zuerstin4=min(which(sec==4));
print(zuerstin4)
  print("************")
  # end of diagnostic output

###################################################################

  # arrival in target 1
  targetsec=zuerstin1

  # duration until arrival in target sector
  q=(targetsec-zuerstrausCS)

  # define vector for distributions
  alldis=c()

  # calculate print distribution for different time slots
  for (jdis in 0:length(sttoffvec)){

    # define relevant time interval
    if (jdis==0) intrvl=1:length(sec) else
intrvl=(zuerstrausCS+sttoffvec[jdis]):(zuerstrausCS+endoffvec[jdis])

    # calculate sector distribution

disxxx=c(sum(sec[intrvl]==1),sum(sec[intrvl]==2),sum(sec[intrvl]==3)
,sum(sec[intrvl]==4))
    alldis=c(alldis,as.vector(disxxx))

    # provide terminal output
    if (jdis==0) {print ("distribution")} else {
      print(paste("distribution from ",sttoffvec[jdis]/60, "sec.
after leaving CS till", endoffvec[jdis]/60, "sec."))}
    print(disxxx)

    # calculate sector transitions
    # initialize vector to collect transition data
    nvec=rep(0,12)
    secmat=expand.grid(1:4,1:4)[c(-1,-6,-11,-16),]
    for (j in 1:(length(sec[intrvl])-1)){
      for (isec in 1:12) if (sec[j]==secmat[isec,1] &
sec[j+1]==secmat[isec,2]) nvec[isec]=nvec[isec]+1
    }

    # terminal output of sector transitions
    if (jdis==0) {print ("distribution")} else {
      print(paste("transitions from ",sttoffvec[jdis]/60, "sec.
after leaving CS till", endoffvec[jdis]/60, "sec."))}
```

```
    # sort output for consisteny with table headings
    ymat[jdis+1,]=nvec[c(4,1,8,5,7,2,10,3,11,6,12,9)]

    # terminal output
    print(ymat[jdis+1,])
    #----


####################################################################
    # Graphical output

####################################################################
    # plot arena
    plot(z[intrvl,id+1], z[intrvl,id+2], pch=10, col=sec[intrvl],
cex=0.5, xlab="x", ylab="y", xlim=c(0,1288), ylim=c(0,996),
main="path 60s", ann=FALSE, axes=FALSE)
    lines(c(cx,cx),c(cy,cy-487)); lines(c(cx,cx),c(cy,cy+487));
lines(c(cx,cx+487*cos(0)),c(cy,cy+487*sin(0)));
lines(c(cx,cx+487*cos(pi)),c(cy,cy+487*sin(pi)))
    if (jdis>0){
      segments(RULx,RULy,ROLx,ROLy)
      segments(RURx,RURy,RORx,RORy)
      segments(RULx,RULy,RURx,RURy)
      segments(ROLx,ROLy,RORx,RORy)
    }
    text(min(z[,id+1]),min(z[,id+2]),paste("fly",i))
    #---

        # define plot title
    if (jdis==0) {mainstr="sector distribution in frames"} else {
      mainstr=paste("sector distribution in frames",sttoffvec[jdis]/
60, "sec. after leaving CS till", endoffvec[jdis]/60, "sec.")}

    # histogramm of sector data
    strmain=
    hist(sec[intrvl], breaks=c(0.5,1.5,2.5,3.5,4.5),
main=paste("fly",i,mainstr),col=c("black", "red", "green", "blue"))

    if (jdis==length(sttoffvec)){
      plot(z[(zuerstrausCS:targetsec), id+1],
z[(zuerstrausCS:targetsec), id+2], pch=10, col="black", cex=0.5,
xlab="x", ylab="y", xlim=c(0,1288), ylim=c(0,996), main=paste("path
to target sec"), ann=FALSE, axes=FALSE)
      lines(c(cx,cx),c(cy,cy-487)); lines(c(cx,cx),c(cy,cy+487));
lines(c(cx,cx+487*cos(0)),c(cy,cy+487*sin(0)));
lines(c(cx,cx+487*cos(pi)),c(cy,cy+487*sin(pi)))
      segments(RULx,RULy,ROLx,ROLy); segments(RURx,RURy,RORx,RORy);
segments(RULx,RULy,RURx,RURy); segments(ROLx,ROLy,RORx,RORy)
      text(min(z[,id+1]),min(z[,id+2]),paste("fly",i))
    }


    # end of graphical output
```

```
####################################################################
  }

  # collect data for output data frame
  d<-rbind(d,c(alldis, ymat[1,], ymat[2,], ymat[3,], ymat[4,],
ymat[5,], ymat[6,], ymat[7,], zuerstin1, zuerstin2, zuerstin3,
zuerstin4,q))
}

# close pdf file
dev.off()

# reset R output
sink()

# write output data frame to file
# define row names in dataout
rownames(d)<-c("fly0")

# define column names in dataout
colnames(d)<-c("Distribution Sec1", "Distribution Sec2",
"Distribution Sec3", "Distribution Sec4","Distribution Sec1 30s",
"Distribution Sec2 30s", "Distribution Sec3 30s", "Distribution Sec4
30s","Distribution Sec1 60s", "Distribution Sec2 60s", "Distribution
Sec3 60s", "Distribution Sec4 60s","Distribution Sec1 90s",
"Distribution Sec2 90s", "Distribution Sec3 90s", "Distribution Sec4
90s","Distribution Sec1 120s", "Distribution Sec2 120s",
"Distribution Sec3 120s", "Distribution Sec4 120s","Distribution
Sec1 30s_60s", "Distribution Sec2 30s_60s", "Distribution Sec3
30s_60s", "Distribution Sec4 30s_60s","Distribution Sec1 30s_90s",
"Distribution Sec2 30s_90s", "Distribution Sec3 30s_90s",
"Distribution Sec4 30s_90s", "n12", "n21", "n23", "n32", "n13",
"n31","n14", "n41", "n24", "n42", "n34", "n43", "n12 30s", "n21
30s", "n23 30s", "n32 30s", "n13 30s", "n31 30s","n14 30s", "n41
30s", "n24 30s", "n42 30s", "n34 30s", "n43 30s","n12 60s", "n21
60s", "n23 60s", "n32 60s", "n13 60s", "n31 60s","n14 60s", "n41
60s", "n24 60s", "n42 60s", "n34 60s", "n43 60s", "n12 90s", "n21
90s", "n23 90s", "n32 90s", "n13 90s", "n31 90s","n14 90s", "n41
90s", "n24 90s", "n42 90s", "n34 90s", "n43 90s","n12 120s", "n21
120s", "n23 120s", "n32 120s", "n13 120s", "n31 120s","n14 120s",
"n41 120s", "n24 120s", "n42 120s", "n34 120s", "n43 120s","n12
30-60s", "n21 30-60s", "n23 30-60s", "n32 30-60s", "n13 30-60s",
"n31 30-60s","n14 30-60s", "n41 30-60s", "n24 30-60s", "n42 30-60s",
"n34 30-60s", "n43 30-60s","n12 30-90s", "n21 30-90s", "n23 30-90s",
"n32 30-90s", "n13 30-90s", "n31 30-90s","n14 30-90s", "n41 30-90s",
"n24 30-90s", "n42 30-90s", "n34 30-90s", "n43 30-90s","First in 1",
"First in 2", "First in 3", "First in 4", "frames to target sec")

# write data to file
write.csv(d,
file="RDataOutput.csv")#########################################

#-----------------
```
